# Subclinical hypothyroidism or isolated high TSH in hospitalized patients with chronic heart-failure and chronic renal-failure

**DOI:** 10.1038/s41598-021-90193-8

**Published:** 2021-05-26

**Authors:** Amir Bashkin, Wagde Abu Saleh, Mona Shehadeh, Lea Even, Ohad Ronen

**Affiliations:** 1 Department of Endocrinology, Galilee Medical Center, POB 21, 2210001 Nahariya, Israel; 2 Department of Geriatric Medicine, Galilee Medical Center, Nahariya, Israel; 3 Department of Biochemistry and Endocrinology Laboratory, Galilee Medical Center, Nahariya, Israel; 4 Department of Pediatrics, Galilee Medical Center, Nahariya, Israel; 5 Department of Otolaryngology Head and Neck Surgery, Galilee Medical Center, Nahariya, Israel; 6 Azrieli Faculty of Medicine, Bar-Ilan University, Safed, Israel

**Keywords:** Biochemistry, Diseases, Endocrinology

## Abstract

Sub-clinical hypothyroidism (SCH) is common in heart failure (HF) and advanced renal failure (RF), but it is unclear whether there is a thyroid disease or a transient increase in TSH level. This is a retrospective study of hospitalized patients in medical departments. All patients with SCH and a TSH level up to less than 12 mIU/L were identified. Those who had at least one recurring admission within at least 6 months were included. A change in thyroid function during the last re-admission was determined and classified as an improvement, no change, or worsening of thyroid function. Overall, 126 cases of SCH met the inclusion criteria for re-admission**.** Analysis of the most recent hospitalization showed that in 100 (79.4%) patients thyroid function improved, in 15 (11.9%) patients thyroid function remained unchanged and only in 11 (8.7%) patients did thyroid function worsen. In most cases, worsening of hypothyroidism was determined by initiation of a low dose levothyroxine treatment. Of the 126 participants, 43 (34.1%) and 22 (17.5%) had a diagnosis of HF and RF (CKD stages 4 and 5), respectively. There was no association between HF or advanced RF and worsening of SCH. No association was found between worsening of hypothyroidism and gender, age, TSH, or creatinine levels in the first hospitalization. A borderline association between elevated CRP levels at first hospitalization and hypothyroidism worsening was found (p = 0.066). Mildly elevated TSH in hospitalized patients with HF and advanced RF is transient and most probably not related to thyroid disease and not associated with age or gender.

## Introduction

The incidence of thyroid dysfunction in hospitalized patients is high, mainly due to the effect of acute disease (non-thyroidal illness—NTIS) and medications^1^. The best laboratory assessment of thyroid function is a serum thyroid-stimulating hormone test if the hypothalamic-pituitary-thyroid axis is intact^2^. Subclinical hypothyroidism (SCH) is defined as a serum thyroid-stimulating hormone (TSH) level above the upper reference limit with a normal level of serum free thyroxine (FT4); 90% of patients with SCH have a TSH level < 10 mIU/L^3^. SCH is considered an initial state of hypothyroidism that develops into an overt state^2,4^. Acute disease is usually accompanied by a decrease in FT3 levels and, in more severe disease, is accompanied by a decrease in TSH and FT4 levels^5^. During the recovery phase from NTIS there may be an increase in TSH levels^6^, even up to 20 mIU/L^7^. Our group recently published a study finding that 3.1% of hospitalized patients admitted to the Medicine Division in our medical center had high TSH levels^8^. Isolated TSH elevations in chronic nonthyroidal illnesses have been attributed to mild hypothyroidism^9^ and it is unclear whether levothyroxine treatment is necessary^10^. In a small percentage of the population an increase in TSH levels occurs without an accompanying decline in FT4, yet in about half of these patients TSH levels return to normal during follow-up^11^. Age-related increase in TSH levels with no decline in FT4 levels is a commonly-known phenomenon, so SCH is particularly common in the elderly, where it can be seen in over 10% of the population over the age of 60^12^. An increase in TSH levels without a decline in FT4 levels is also common in obesity^13^. In depressed inpatients the prevalence of SCH in females (14.6%) was approximately two times that of males, and high-BMI females were at high risk to develop SCH^14^.

Hypothyroidism is associated with remarkable alteration in cardiac contractility and output, myocardial oxygen consumption, vascular resistance, blood pressure and electrophysiological conduction^15^. It contributes to hypercholesterolemia, diastolic hypertension, statin myopathy, and left ventricular diastolic dysfunction, leading to heart failure with preserved ejection fraction^16^. Hypothyroidism may affect renal function through direct mechanisms on glomerular and tubular functions and also indirectly through modifications in cardiac and vascular function and derangements in the renin-angiotensin system^17^. SCH is common in various chronic diseases including heart failure and end-stage renal failure, but it is unclear whether there is a thyroid disease or an isolated increase in TSH level^18,19^. In a prospective cross-sectional study that included 452 hospitalized patients with systolic heart failure, 20 of these patients were diagnosed with sub-clinical hypothyroidism. After a 6-month follow-up, thyroid function was found to be normal in 15 patients, 5 patients were left with SCH, and no patient developed overt hypothyroidism^20^. In a cross-sectional study that included 16 hemodialysis patients, an increase in TSH levels was found in 4 of the patients; all resolved during a 14-month follow-up^21^. In patients on hemodialysis, higher serum TSH levels are associated with impaired health-related quality of life^22^. In Japanese patients CKD was associated with SCH, as was age; however, gender was not associated with hypothyroidism among CKD patients^23^. For all the reasons mentioned above, slightly increased TSH and FT4 levels are within the norm in patients hospitalized with heart or renal failure; it is impossible to know whether the occurrence is an isolated increase in TSH level without thyroid disease, or whether it is SCH.

The purpose of this work was to examine the association between SCH or an isolated increase in TSH level and the risk of hypothyroidism deterioration in a recurring admission. In addition, we wished to examine whether heart or renal failure are risk factors for thyroid disease that will be diagnosed over time in worsening of hypothyroidism.

## Methods

This was a retrospective study analyzing existing data of hospitalized patients admitted to the Medicine Division of our medical center for any reason. During the study period, TSH level was measured for all patients as part of the admission profile after an overnight fast, and if TSH level was found to be increased, then FT4 level was also measured. The study included all patients hospitalized in the internal medicine departments as well as Geriatrics, Cardiology, Nephrology and Neurology. Patients in Intensive Care Units and Oncology departments were not included. The data were collected from electronic medical records. All patients over the age of 18 who were hospitalized between the years 2013–2016 with subclinical hypothyroidism and a TSH level above normal (4.95 mIU/L) and up to < 12 mIU/L were identified. This TSH cutoff was chosen because we wanted to include the highest TSH that is not associated with deterioration to overt hypothyroidism. Next, all patients who had at least one recurring admission within at least 6 months were included. In addition, all patients who had a recurring admission within at least 1 year were included. From each hospitalization, laboratory data were collected on admission including thyroid function tests, C-reactive protein (CRP), and creatinine. The blood was drawn in the morning after an overnight fast. In addition, patients with congestive heart failure (systolic or diastolic) and advanced chronic renal failure with a calculated glomerular filtration rate (eGFR) less than 30 ml/min/1.72 m^2^ (chronic kidney disease, CKD, stages 4 and 5) were identified. Chronic renal failure was determined according to chronic renal failure diagnosis in the patient history and based on eGFR of less than 30 ml/min/1.72 m^2^. Heart failure diagnosis was based on patient history diseases list and was verified according to the laboratory and imaging tests, as well as drug therapy. On recurrent admissions it was determined whether the thyroid function tests had improved, had not changed or had worsened.

Patients with thyroid disorders who needed pharmacologic treatment in the first hospitalization, or with a thyroid function test that was performed after transfer from another department, were excluded from the study as well as patients who took medications with a significant effect on thyroid function (in any hospitalizations) including levothyroxine, anti-thyroid drugs, amiodarone, systemic corticosteroids, phenytoin, carbamazepine, rifampicin, lithium, interferon, interelekin-2, and tyrosine kinase inhibitors.

A change in thyroid function during the last re-admission was determined according to the following: TSH level within the normal level was defined as an improvement, an increased TSH level but less than 12 mIU/L was defined as no change, and a TSH level over 12 mIU/L or initiation of levothyroxine treatment was defined as worsening of thyroid function. We included all TSH levels in readmission cases, and did not limit them to 12 mUI/L as had been done in the inclusion criterion during the first admission.

In order to examine the association between background diseases (heart failure and chronic renal failure) and gender, and worsening of SCH, we considered the cases with improved or unchanged TSH on re-admission as one group and compared them to patients who had worsening in hypothyroidism on re-admission. The relationship between age, TSH and CRP levels on first admission, and worsening of hypothyroidism on re-admission was analyzed twice. First, the data from all groups of TSH on re-admission (improvement, no change, and worsening) were analyzed, and again the groups were divided into those who had worsening in TSH or initiation of thyroxine treatment, and those who did not worsen.

TSH and FT4 levels were measured using chemiluminescent microparticle immunoassay in the Architect i2000SR (Abbott Diagnostics, Illinois, USA), in which the normal ranges for TSH were 0.35–4.98 mIU/L, for FT4 9–19 pmol/L (0.7–1.48 ng/dL) and for FT3 2.6–5.7 pmol/L (1.71–3.71 pg/ml). CRP levels were analyzed using an immunoturbidimetric assay on the Architect System (Abbot Diagnostics, Illinois, USA) in which the normal range was 0.2–5 mg/L.

### Statistical analysis

Quantitative data were described using averages and standard deviations, median, and range. Qualitative data were described by frequencies and percentages. Comparisons between groups of patients were conducted using Wilcoxon rank sum test for quantitative data and Chi square test, Fisher's exact test for qualitative data or Fisher-Freeman-Halton Exact Test. Logistic regression was used to confirm the independent correlations with hypothyroidism exacerbation. The statistical analysis was performed using SPSS 19, a *p* value less than 0.05 was considered significant.

### Ethical approval

The study was approved by the Galilee Medical Center’ Institutional Review Board (NHR009914). All procedures were carried out according to relevant guidelines.

### Informed consent

Informed consent was waived by the Galilee Medical Center IRB.

## Results

During the years 2013–2016, 90,199 TSH tests were performed in the departments included in the study, some of them were repeated tests but most were carried out as part of the admissions profile.

Overall, 2,116 patients with a TSH level above the normal range but less than 12 mIU/L and with a normal FT4 level were identified. Of the 2116 hospitalized cases, 126 cases met the inclusion criteria for re-admission. Patients' characteristics are described in Table 1. The median age of the 126 patients was 69.5 years (range 18–97 years), 61.1% were women, and the median TSH level was 6.3 mIU/L (range 5–11.2 mIU/L). Of the 126 cases, 80 had another hospitalization within at least 6 months, and had a median TSH level of 3.2 mIU/L (range 0.6–242.2 mIU/L). Fifty-five patients were re-hospitalized within at least 1 year and had a median TSH level of 2.66 mIU/L (range 0.07–14.3 nIU/L). Nine participants were readmitted after at least 6 months and had a second hospitalization after at least 1 year, therefore, for analysis purposes only the latter hospitalization was considered. Of the 126 participants, 43 (34.1%) had a diagnosis of systolic and/or diastolic heart failure and 22 (17.5%) had a diagnosis of chronic renal failure (CKD stages 4 and 5).

**Table 1:** Baseline characteristics in first hospitalization.

Characteristic	Mean ± SD, median, (range)
Age (years)	67.8 ± 16.7, 69.5 (18–97)
TSH (IU/L)	6.6 ± 1.3, 6.3 (5–11.2)
FT4 (pmol/L)	12.9 ± 2.6, 12.9 (9–19.1)
CRP (mg/L)	34.5 ± 55.1, 10.6 (0.6–288.9)
Creatinine (mg/dl)	1.4 ± 1.2, 1.0 (0.5–10.3)

*FT4* free T4, *CRP* C-reactive protein.

Analysis of the most recent hospitalization of all 126 cases showed that in 100 (79.4%) patients thyroid function improved; that is, the TSH level normalized. In 15 (11.9%) patients thyroid function remained unchanged and only in 11 (8.7%) patients did thyroid function worsen. Of the 11 cases that had worsening of thyroid function, seven had an initial levothyroxine treatment prior to hospitalization in an ambulatory setting and in three levothyroxine was initiated during hospitalization. In one case, treatment with levothyroxine was initiated prior to hospitalization, and in re-admission there was another significant elevation in TSH level. Of the 8 cases in which levothyroxine treatment was initiated before hospitalization, five were treated with a low dose of levothyroxine (50 µg per day). Most of these patients had additional hospitalizations, no alteration in levothyroxine dose was done, and no examination of treatment necessity was done.

Patients' reason for admission stratified between admissions is represented in Table 2. No significant differences were found between the groups.

**Table 2:** Patients' reason for admission stratified between admissions.

Reason for admission	1st admission N (%)	2nd admission N (%)	3rd admission N (%)	P value
Gender—female	77 (61.1%)	44 (55.0%)	39 (70.9%)	0.178*
Age (years), mean ± SD, median (range)	67.8 ± 16.7, 69.5 (18–97)	69.5 ± 16.8, 71.9 (18–97)	70.3 ± 14.2, 70.8 (41–93)	0.480^‡^
Infection	35 (28%)	24 (30%)	19 (35%)	0.655*
Cardiovascular	11 (9%)	3 (4%)	2 (4%)	0.309^†^
Heart failure	8 (6%)	10 (13%)	5 (9%)	0.306^†^
Obstructive lung disease	3 (2%)	6 (8%)	4 (7%)	0.150^†^
Chest pain	22 (17%)	9 (11%)	10 (18%)	0.426*
Other	37 (29%)	25 (31%)	12 (22%)	0.469*
Renal failure and electrolyte abnormality	4 (3%)	3 (4%)	2 (4%)	1.000^†^
Arrhythmia	6 (5%)	0 (0%)	1 (2%)	0.111^†^
Overall	126	80	55	

*2-sided Pearson Chi Square.

^‡^2-sided Independent Sample t-Test.

^†^2-sided Fisher-Freeman-Halton Exact Test.

The association between heart and/or chronic renal failure and gender and worsening of SCH is presented in Table 3. When the participants were divided into two groups, with and without worsening in hypothyroidism, there was no association between heart and/or chronic renal failure and worsening of SCH (*p* = 1). There was no difference in gender prevalence between these two groups of patients.

**Table 3:** Heart and renal failure and hypothyroidism worsening between hospitalizations.

	Hypothyroidism between hospitalizations		*p* value*
	Worsening hypothyroidism	Thyroid function improved or remained unchanged	
CHF n (%)	4/43 (9.3%)	7/83 (8.4%)	1
CRF n (%)	2/22(9.1%)	9/104 (8.7%)	1
Female n (%)	7/11 (63.6%)	70/115 (60.9%)	1

*CHF* congestive heart failure, *CRF* chronic renal failure.

*Fisher's Exact Test.

The association between age, TSH, CRP, and creatinine levels during first hospitalization and worsening of hypothyroidism, when cases were divided into 3 groups (worsening, no change or improvement in TSH levels) is shown in Table 4. No association was found between worsening of hypothyroidism and age (*p* = 0.62), TSH levels (*p* = 0.28), CRP levels (*p* = 0.17), or creatinine levels (*p* = 0.74) for all 126 cases.

**Table 4:** Hypothyroidism worsening between hospitalizations and clinical characteristics in the first hospitalization.

Characteristics	Hypothyroidism between hospitalizations		P value*
	Worsening (n = 11)	Improved or unchanged (p = 115)	
Age (years) mean ± SD, median (range)	70.5 ± 13.2, 74 (46–90)	67.5 ± 17, 69 (18–97)	0.62
TSH (IU/L) mean ± SD, median (range)	6.2 ± 1.0, 6.0 (5–7.81)	6.7** ± **1.3, 6.3 (5.0–11.2)	0.28
CRP (mg/L) mean ± SD, median (range)	69.8 ± 74.9, 29.1 (1.8–209.1)	31.1± 52.0, 9.9 (0.6–288.9)	0.17
Creatinine (mg/dl) mean ± SD, median (range)	1.4 ± 1.4, 0.8 (0.6–5.3)	1.4 ± 1.2, 1.0 (0.5–10.3)	0.74

*CRP* C-reactive protein, *CRE* creatinine.

*Kruskal Wallis Test.

*Wilcoxon sum rank test

Table 5 presents the association when the cases were divided into two groups: those with worsening hypothyroidism and those whose hypothyroidism did not worsen. A borderline association between CRP levels at first visit and hypothyroidism worsening was found (*p* = 0.066).

**Table 5:** Worsening hypothyroidism between hospitalizations and clinical factors in the first hospitalization.

Characteristics	Hypothyroidism between hospitalizations		P value*
	Worsening (n = 11)	No-worsening (p = 115)	
Age, years mean ± SD, median (range)	74 (46–90)	69 (18–97)	0.677
TSH, IU/L, mean ± SD, median (range)	6 (5–7.81)	6.3 (5–11.2)	0.262
CRP (mg/L), mean ± SD, median (range)	29.1 (1.8–209.1)	9.9 (0.6–288.9)	0.066

Wilcoxon sum rank test.

*CRP* C-reactive protein.

*Wilcoxon sum rank test

In patients who had worsening in hypothyroidism, CRP levels were higher in the first hospitalization, with a median of 29.1 mg/L and a range of 1.8–209.1 mg/L compared to a median of 9.9 mg/L and a range of 0.6–288.9 mg/L in patients with no worsening in hypothyroidism. No association was found between the increase in CRP levels during the first and last hospitalization and improvement in hypothyroidism (*p* = 0.68). In addition, there was no association between a decrease in CRP levels during the first and last hospitalization and worsening of hypothyroidism (*p* = 0.37, Wilcoxon Signed Ranks Test).

Only CRP was found to influence hypothyroidism worsening on multivariate analysis using backward logistic regression (p = 0.039), albeit with very little impact (OR 1.009, 95%CI 1.000–1.017).

## Discussion

In most (91.3%) patients hospitalized with SCH or isolated high TSH level and TSH level up to 12 mIU/L, and who were re-admitted within at least 6 months, we found no deterioration in hypothyroidism. During the re-admission thyroid function improved in 79.4%, was unchanged in 11.9% and worsened in 8.7% of cases. In most cases, worsening of hypothyroidism was determined by initiation of levothyroxine treatment and it was impossible to determine whether the treatment was justified.

Of the 8 cases in which levothyroxine thyroxine was initiated prior to re-admission, five were euthyroid under a minimal dose of 50 µg per day and none had reduced free T_4_ levels, so the treatment may have been unnecessary. Of the cases included in the study, 34.1% had heart failure and 17.5% had chronic renal failure. In patients hospitalized with SCH no association was found between heart and/or renal failure and worsening in hypothyroidism (*p* = 1). Primary hypothyroidism in countries with no iodine-deficiency is mainly due to Hashimoto's thyroiditis which is much more prevalent in women than men^4^. Risk factors for transition from subclinical to overt hypothyroidism are the same risk factors as in Hashimoto's disease including advanced age, female gender, high TSH and positive anti-TPO (thyroid peroxidase antibody)^2,4^. In our study, we did not find an association between the worsening of subclinical hypothyroidism and gender (*p* = 1), age (*p* = 0.677), or TSH level during the first hospitalization (*p* = 0.262). This finding decreases the probability that the increased TSH in the first hospitalization was due to thyroid disease. Isolated mild TSH increase in congestive heart failure patients was associated with increased incidence of heart failure exacerbation^24^. The association between CRP levels in the first hospitalization and worsening of hypothyroidism had a borderline statistical significance (p = 0.066). In patients who had worsening hypothyroidism, CRP levels were higher than in patients with no worsening (median 29.1 mg/L, 1.8–209.1 mg/L compared to median 9.9 mg/L, 0.6–288.9 mg/L, *p* = 0.066). In patients with very high CRP levels the TSH is suppressed due to severe non-thyroidal illness, therefore it is likely that the real TSH level is even higher. In addition, treatment with systemic corticosteroids in hospitalized patients, which also decreases the TSH level, was common in our cohort.

### Limitations and strengths

Our patient sample was relatively large compared to other studies that were conducted on the subject and our study includes a long follow-up period. The study was conducted in one medical center and therefore it is based on one assay, although there are known differences between assays^3^.

We did not have FT3 values for most patients. We believe that in case of an increase in TSH levels it is recommended to evaluate FT4. FT3 level is of less importance for the diagnosis of hypothyroidism, since the deiodinase that converts T4 to T3 is hyperactivated in this situation, and therefore the TSH deficiency is better expressed in the level of FT4 than in FT3 levels. For this reason, we did not routinely measure FT3 in these patients.

Other data unavailable to us were natriuretic peptide levels as well as creatinine and phosphorus in the different patients’ groups.

Since our study was of a retrospective design, it is impossible to know the considerations for levothyroxine treatment initiation, as well as anti TPO and anti TG antibody levels. In addition, we did not have any data regarding the change in patients’ symptoms nor the results of thyroid ultrasound. A prospective study that would collect those parameters has the potential to overcome these limitations.

## Conclusions

Similar to the comprehensive review by Kaptein et al.^18^, our conclusion is that an increase in TSH levels in patients with heart and/or renal failure is unrelated to thyroid disease. Therefore, we did not find an association between thyroid worsening dysfunction and gender or age. It is much more likely that the observed increase in TSH levels is related to other causes mentioned earlier, such as advanced age, obesity and recovering from acute disease.

## Data Availability

All applications for additional data sharing will be considered by the authors upon request.

## Abbreviations

T3: triiodothyronine
T4: Thyroxine
NTIS: Non-thyroidal illness syndrome
CRP: C-reactive protein
TSH: Thyroid stimulating hormone
FT3: Free T3
FT4: Free T4
CKD: Chronic kidney disease
TPO: Thyroid peroxidase antibody
